# Palliative care, its need and role in cancer patients, in the Indian scenario

**DOI:** 10.4103/0971-5851.64260

**Published:** 2009

**Authors:** Rajiv Saini, Santosh Saini, Sugandha

**Affiliations:** Departments of Periodontology and Oral Implantology, Rural Dental College-Loni, Maharashtra, India. E-mail: drperiodontist@yahoo.co.in; 1 *Departments of Microbiology, Rural Dental College-Loni, Maharashtra, India*; 2 *Departments of Prosthodontics, Rural Dental College-Loni, Maharashtra, India*

Sir,

The World Health Organization (WHO) defines palliative care as the active total care of the patient, whose disease is not responsive to curative treatment. Control of pain, other symptoms, and the psychological, social, and spiritual problems are paramount. The goal of palliative treatment is the achievement of the best possible quality of life (QOL) for patients and their families.[1] Palliative care is an imperative need worldwide for people with cancer, Human Immunodeficiency Virus / Acquired Immunodeficiency Virus, and other life-threatening illness. Palliative care is a relatively new and still emerging specialty area of practice. The situation is particularly critical in less-developed countries, where patients are diagnosed in their advanced stages and there is limited access to prevention and treatment services. Prognostic paralysis has been described, whereby, clinicians of patients with uncertain illness trajectories prevaricate when considering end of life issues.[2] Health, social, and palliative care services are continuing to fail many people with progressive chronic illnesses, in whom death may be approaching, reflecting a failure to think proactively and holistically about their care.[3] Developing and promoting all the eras of palliative care in a community setup is a must for the proper palliative care of the needy. Palliative care treatment should focus on the improvement of the quality of life instead of straining the curative treatment approach. In palliative medicine an interdisciplinary approach is inevitable and imperative. The problems contributing to suffering in incurable diseases includes pain, anorexia, nausea, fungating wounds, disfigurement, breathlessness, loss of social roles, social isolation, dependency, personality changes, sadness, depression, anger, fatigue, anxiety, financial difficulties, bowel obstruction, oral / mouth problems, insomnia, diarrhea, confusion, delirium, and so on.

Community need and the role of palliative care - As per the WHO figures, around one million new cases of cancer are diagnosed each year. About one-third of the patients are being treated for cancer and three-fourth is in terminal stages, and have pain. There is a need to increase the awareness among the community with regard to palliative care and its role at the end stage of any chronic illness. Greater awareness of palliative care should make it easier for professionals to combine active treatment and a supportive approach. People with disabling, progressive illnesses expect active care, but they also seek comfort, control, and dignity. The barriers to effective communication about emotional and end-of-life issues are well recognized.[4]

Palliative care: Indian scenario - The message of palliative care has become a movement in several parts of India in a short span of time. The past two decades have seen palpable changes in the mindset of healthcare providers, and policy makers with respect to the urgency of providing palliative care.[5] Every hour more than 60 patients die in India from cancer and in pain. Moreover, with a population of over a billion, spread over a vast geo-political mosaic, the reach and reliability of palliative care programs may appear staggering and insurmountable.[5] Furthermore, the treatment should focus on the improvement of quality of life instead of straining the curative treatment approach; in palliative medicine an interdisciplinary approach is inevitable and essential.[6] The current training in palliative oncology seems inadequate in terms of providing comprehensive care, caregivers are often ill-prepared for confronting the experience of death, and providing supportive care and attention to a patient with advanced cancer.[7] To overpower this hurdle in future, an appropriate patient follow-up and care delivery system must be carefully and logically structured by providing in-house pain clinics and establishing hospices in close proximity to the comprehensive cancer care centers, with dedicated beds and logistic support, which can improve the compliance of patients.[7] Broadening the course of palliative care by providing education to clinicians, patients, and families with regard to the elements and appropriateness of palliative care, can also help. A process that will allow providers to identify and assess patients who would benefit from palliative care services must be developed. This process must include the use of a screening tool that utilizes the seven domains, by developing a process for timely referral of patients with a progressive, debilitating disease for palliative care consultation.
